# A New Species of the Genus *Boulenophrys* (Anura, Megophryidae) from Southern Hunan Province, Central China †

**DOI:** 10.3390/ani15030440

**Published:** 2025-02-05

**Authors:** Bei Xiao, Jiayan Xi, Shengchao Shi, Hui Li, Leqiang Zhu, Ayinuer Maimaiti, Yalan Xu, Shunhua Liao, Bin Wang, Xiaoyang Mo

**Affiliations:** 1 Vertebrate Zoology Laboratory, Hunan Normal University, Changsha 410081, Chinabiomen@foxmail.com (S.S.);; 2 Chengdu Institute of Biology, Chinese Academy of Sciences, Chengdu 610041, China; 3 Hubei Engineering Research Center for Protection and Utilization of Special Biological Resources in the Hanjiang River Basin, School of Life Science, Jianghan University, Wuhan 430056, China; 4 College of Biological and Food Engineering, Huaihua University, Huaihua 418000, China; 5 Dupangling National Nature Reserve, Yongzhou 425300, China

**Keywords:** Asian horned toads, taxonomy, Hunan Province, *Boulenophrys dupanglingensis* sp. nov

## Abstract

A new species of Asian horned toad, *Boulenophrys,* is described from Yongzhou City, Hunan Province, China. The species is a phylogenetically sister to *B. yunkaiensis,* based on 16S rRNA and *COI* genes. The new species differs from its congeners, possessing the following combination of characters: (1) moderate body size: SVL 37.6–40.2 mm (38.9 ± 1.3, *n* = 7) in adult males and SVL 41.8–45.9 mm (43.6 ± 2.1, *n* = 3) in adult females; (2) tympanum boundary clear: TD/ED 0.48–0.57 in males and 0.47–0.57 in females; (3) the presence of a small horn-like tubercle at the edge of the upper eyelid; (4) vomerine ridge present and vomerine teeth absent; (5) margin of tongue rounded, not notched posteriorly; (6) rough dorsal skin: a discontinuous “V”-shaped ridge with two discontinuous dorsolateral ridges on two sides on the back, dense tubercles on the skin of the ventral surface of the dorsal shank and thigh, and spiny tubercles surrounding the cloaca; (7) slender hindlimbs with heels overlapping when the flexed hindlimbs are held at right angles to the body axis; tibio-tarsal articulation reaching forward between anterior margin of tympanum and posterior corner of eye when leg stretched forward; (8) relative finger length IV < II < I < III, with a subarticular tubercle present at the base of each finger; (9) distinct supernumerary tubercles below the base of I and II toes; (10) toes without lateral fringes and with rudimentary webbing (webbing formula: I1 − 1-II1 − 2-III2 − 3IV3- − 2V).

## Simple Summary

The genus *Boulenophrys* Fei, Ye & Jiang, 2016, consists of 69 recognized species and is known for its endemism and diversity. This study describes a new species from Yongzhou City, Southern Hunan Province, Central China: *Boulenophrys dupanglingensis* sp. nov. The species presents a monophyletic lineage that differs from its sister species *B. yunkaiensis*, possessing relatively shorter shanks, distinct supernumerary tubercles below the base of the I and II toes, rough dorsal skin with dense granules, and several tubercles on the flanks.

## 1. Introduction

The subfamily Megophryinae is endemic to Asia, with a distribution spanning the Himalayas, northeastern India, southern China, and extending southwards to the islands of Southeast Asia [1,2,3]. The classification of Megophryinae has been a subject of debate for decades. Molecular phylogenetic studies consistently indicate that it is a monophyletic group [4,5,6]. However, the generic relationships and divisions within this subfamily have went through multiple alternations in recent years [4,5,6,7,8,9,10]. Lyu et al. proposed a new classification system, identifying ten genera: *Atympanophrys* Tian and Hu, 1983; *Brachytarsophrys* Tian and Hu, 1983; *Grillitschia* Dubois, Ohler, and Pyron, 2021; *Sarawakiphrys* Lyu and Wang, 2023; *Jingophrys* Lyu and Wang, 2023; *Xenophrys* Günther, 1864; *Megophrys* Kuhl and Van Hasselt, 1822; *Pelobatrachus* Beddard, 1907; *Ophryophryne* Boulenger, 1903; *Boulenophrys* Fei, Ye, and Jiang, 2016 [7,10,11,12,13,14,15,16]. Among these, *Boulenophrys* is the most diverse genus, comprising 69 recognized species to date (Table S1).

Dupangling National Nature Reserve is located in the southwest of Yongzhou City, southern Hunan Province, Central China, within the Nanling Mountains. During herpetological surveys conducted in 2016 and 2022, we discovered a species of *Boulenophrys* from Dupangling National Nature Reserve in Daoxian County and Jiangyong County and Yongzhou County of Yongzhou city. The genus was diagnosed based on the diagnostic characters by Lyu et al. [10], these specimens were identified as belonging to the genus *Boulenophrys*. However, due to a unique combination of morphological characteristics, they could not be classified into any known species. Further molecular phylogenetic analysis revealed that these specimens represent a separate evolutionary lineage. Accordingly, we formally describe them here as a new species within the genus *Boulenophrys*.

## 2. Materials and Methods

### 2.1. Sampling

**Figure 1:**
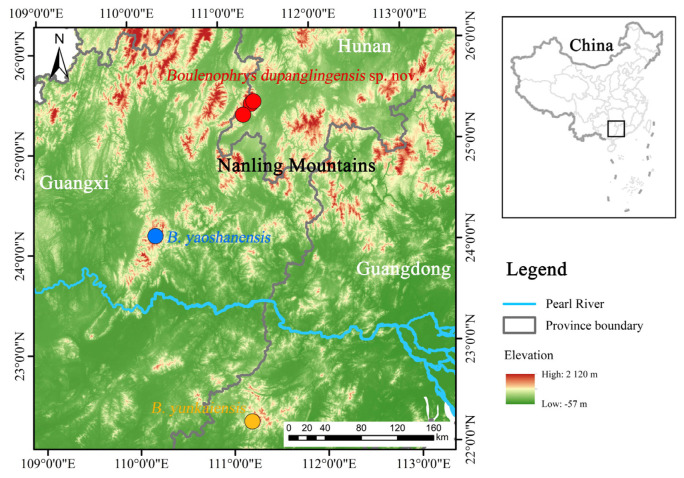
Distribution map of *Boulenophrys dupanglingensis* sp. nov. and the type localities of two phylogenetically close species.

Field surveys were carried out at Dupangling National Nature Reserve, Hunan Province, China (Figure 1), in May 2016 and April 2022. A total of ten specimens were newly collected. Initially, nine specimens were fixed in 10% formalin and later transferred to 75% ethanol for long-term storage. Before formalin fixation, liver tissues from four specimens were sampled and stored in 95% ethanol for DNA extraction. Nine specimens were deposited in the Animal Museum of Life Sciences College of Hunan Normal University (HNNU), Changsha City, Hunan Province, China, while one specimen was stored in 75% ethanol and placed in Chengdu Institute of Biology (CIB), Chinese Academy of Sciences, Chengdu City, Sichuan Province, China.

### 2.2. Molecular and Phylogenetic Analyses

Genomic DNA was extracted from liver tissues preserved in 95% ethanol using the TSINGKE DNA extraction kit. For sequencing, mitochondrial genes, specifically the partial 16S ribosomal RNA gene (16S rRNA) and partial cytochrome c oxidase 1 gene (*COI*), were targeted in four samples (HUNU 22SA05, HUNU 22SA07, HUNU 22SA08, and CIB 2016050802). Polymerase Chain Reaction (PCR) amplifications were conducted following Liu et al. [5]. The PCR products were sequenced using an ABI 3730 XL Genetic Analyzer at Sangon Biotech Co., Ltd. (Shanghai, China), and the obtained sequences were deposited in GenBank.

For the phylogenetic analyses, sequences were obtained from GenBank, including 68 known *Boulenophrys* species (except *B.shuichengensis,* for which no sequence information is available) and 2 outgroup species (*Xenophrys glandulosa* and *X. mangshanensis*) (Table S2). All sequences were aligned using the Clustal W algorithm with default parameters [17] and then trimmed using the partial gap deletion option in MEGA 11 [18]. Gaps in highly variable regions were removed. The best-fit evolutionary model (GTR+G+I) was determined using PartitionFinder v. 2.1.1 based on the AIC criterion [19]. Phylogenetic analyses were conducted using Bayesian inference (BI) with MrBayes 3.2 [20], and maximum likelihood (ML) trees were generated using PhyML v. 3.0 [21]. The BI analysis ran for 20,000,000 generations, with samples taken every 1000 generations, discarding the first 25% as burn-in, resulting in a potential scale reduction factor (PSRF) of <0.005. For the ML analysis, a bootstrap consensus tree was created from 1000 replicates.

### 2.3. Morphological Analysis

The morphological character definitions follow those established by Lyu et al. [10]. The following measurements were taken with digital calipers, with accuracy to the nearest 0.1 mm: SVL, snout–vent length (from the tip of the snout to the posterior of the vent); HDL, head length (from the snout tip to jaw articulation); HDW, head width (at the jaw commissure); SNT, snout length (from the snout tip to the anterior corner of the eye); IND, internasal distance (distance between nares); IOD, interorbital distance (minimum distance between the upper eyelids); ED, eye diameter (from the anterior corner to the posterior corner of the eye); TD, tympanum diameter (horizontal diameter of the tympanum); TED, tympanum–eye distance (from the anterior edge of the tympanum to the posterior corner of the eye); HND, hand length (from the proximal border of the outer palmar tubercle to the tip of digit III); RAD, radio–ulna length (from the flexed elbow to the proximal border of the outer palmar tubercle); FTL, foot length (from the distal end of the shank to the tip of digit IV); TIB, tibial length (from the outer surface of the flexed knee to the heel). The webbing was expressed using the webbed formula [22], with Roman numerals indicating fingers and toes. Sex determination was based on the direct observation of calls or the presence of internal vocal sac openings in males. The presence or absence of nuptial pads was examined under a dissecting microscope.

## 3. Results

### 3.1. Molecular Phylogenetic Analyses

**Figure 2:**
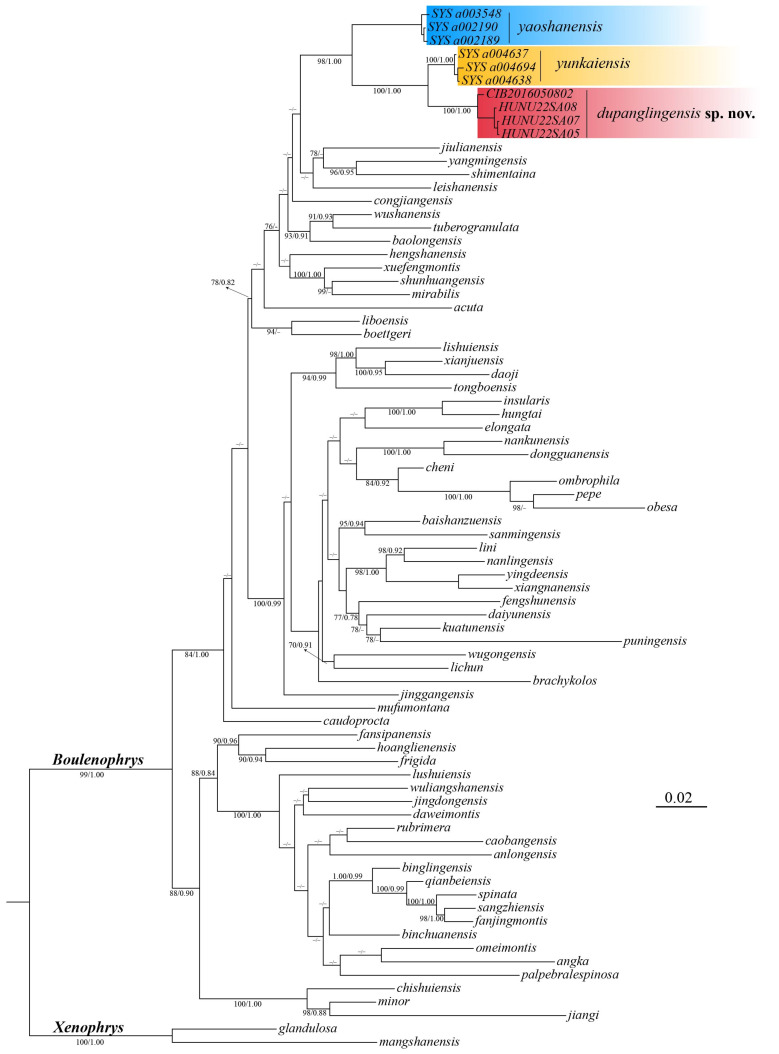
Bayesian topology is based on the partial DNA sequences of the mitochondrial 16S rRNA and *COI* genes, with bootstrap support (BS)/Bayesian posterior probabilities (BPP) displayed at the tree nodes. A dash (‘−’) indicates BS value below 70 or BPP value below 90.

A total of 540 base pairs (bps) of the 16S gene and 636 bps of the *COI* gene were concatenated into a single 1196 bp sequence. Phylogenetic analyses using both ML and BI methods yielded nearly identical results, with strong support for the major nodes in both tree types. The Bayesian tree is shown in Figure 2, where nodes with ML bootstrap values (BS) ≥ 70 and Bayesian posterior probabilities (BPP) ≥ 0.90 are considered to be strongly supported. In different analyses, the newly collected samples consistently formed a monophyletic group, displaying robust nodal support (BS 100, BPP 1.00). Additionally, based on the *p*-distances in the *COI* gene, genetic divergence among species within the *Boulenophrys* genus ranges from 2.3% to 18.9% (Table S3). The genetic distance between the candidate new species and its most close congener *B.yunkaiensis* is 4.3%, which is among interspecific level. Furthermore, these specimens are distinguishable from other congeners within the genus by a unique combination of morphological characters (see taxonomic account below).

### 3.2. Morphological Comparisons

Morphological comparisons for the candidate new species with other recognized species of the *Boulenophrys* genus are listed in Table 1.

The candidate new species is grouped within the same clade with closed congeners (*B. congjiangensis*, *B. jiulianensis*, *B. leishanensis*, *B. shimentaina*, *B.yangmingensis*, *B.yaoshanensis*, *B.yunkaiensis*) based on molecular analysis, and it further morphologically different from the latters by combination of following characters: (1) moderate body size of SVL 37.6–40.2 mm (*n* = 7) in adult males and SVL 41.8–45.9 mm in adult females (*n* = 3) (vs. smaller species *B. congjiangensis* (28.6–33.4, *n* = 15 in males, 38.4–40.2, *n* = 2 in females), *B. jiulianensis* (30.4–33.9, *n* = 10 in males, 34.1–37.5, *n* = 2 in females), and *B. shimentaina* (25.8–30.6, *n* = 20 in males, 34.5 in female)); (2) absence of lateral fringes on the toes (vs. *B. congjiangensis* and *B. shimentaina*, which have narrow lateral fringes); (3) nuptial pads bearing fine and dense black nuptial spines on the dorsal bases of fingers I and II in breeding adult males (vs. *B.yangmingensis* and *B.yaoshanensis* with villiform black nuptial spines); (4) dense tubercles on ventral shank and thigh, and spiny tubercles surrounding the cloaca (vs. *B.leishanensis* and *B.yaoshanensis*, with relatively smooth ventral skin); (5) unnotched tongue (vs. tongue of *B. jiulianensis* weakly notched posteriorly); (6) absence of vomerine teeth (vs. *B. shimentaina* with vomerine teeth present).

**Figure 3:**
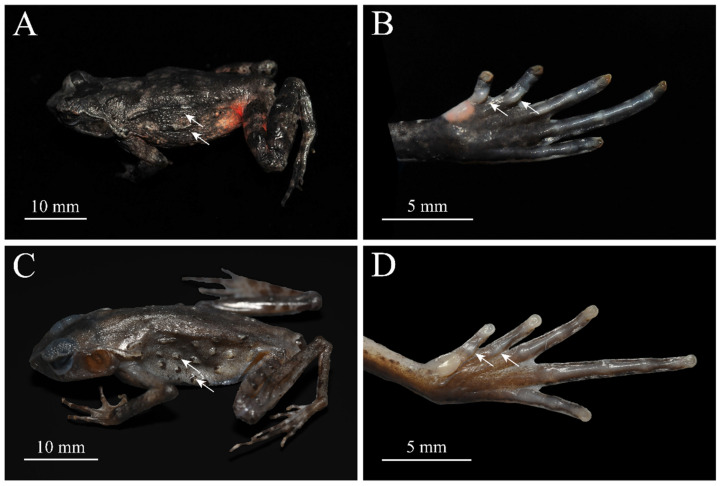
Comparisons of *Boulenophrys dupanglingensis* sp. nov. (CIB2016050802) and *B. yunkaiensis* (SYS a004986) in preservative. White arrows indicate differences between closely related congeners. *Boulenophrys dupanglingensis* sp. nov.: (**A**) dorsolateral view; (**B**) plantar view of foot. *B. yunkaiensis*: (**C**) dorsolateral view; (**D**) plantar view of foot. Photo by Shengchao Shi (**A**,**B**) and Le-Qiang Zhu (**C**,**D**).

The candidate new species is phylogenetically most close to *B. yunkaiensis.* It differs from *B. yunkaiensis* in the following characters: (1) relatively shorter shanks: TIB/SVL 0.37–0.39 (0.38 ± 0.01, *n* = 7) in adult males (vs. TIB/SVL 0.40–0.48, 0.44 ± 0.04, *n* = 3) and 0.32–0.36 in adult females (0.34 ± 0.02, *n* = 3) (vs. TIB/SVL 0.42–0.46 in adult, 0.44 ± 0.02, *n* = 3 for *B. yunkaiensis*); (2) distinct supernumerary tubercles below the base of toes I and II (vs. indistinct tubercles under all toes in *B. yunkaiensis*); (3) rough dorsum skin with several dense small granules on the flanks (vs. sparse larger granules on the flanks) (Figure 3).

The candidate new species can be easily distinguished from 16 other congeners by its relatively longer shanks and overlapping of its heels when the flexed hindlimbs are held at right angles to the body axis, differing from *B*. *acuta*, *B*. *brachykolos*, *B*. *daoji*, *B*. *dongguanensis*, *B*. *fengshunensis*, *B*. *hungtai*, *B. hengshanesis*, *B*. *insularis*, *B. kuatunensis*, *B.lichun*, *B*. *nankunensis*, *B*. *obesa*, *B*. *ombrophila*, *B*. *puningensis*, *B*. *wugongensis* (vs. relatively shorter shanks with the heels not meeting), and *B. xuefengmontis* (vs. just meeting).

The candidate new species can be distinguished from nine congeners by its unnotched tongue, including *B. baolongensis*, *B. binlingensis*, *B. boettgeri*, *B. cheni*, *B. lushuiensis*, *B. minor*, *B.mufumontana*, *B.qianbeiensis*, and *B. sanmingensis* (vs. tongue notched posteriorly). By having a subarticular tubercle present at the base of each finger, it differs from the following seven congeners: *B. angka*, *B. chishuiensis*, *B. leishanensis*, *B. lishuiensis*, *B. tuberogranulatu*, *B. wuliangshanensis*, and *B. yaoshanensis* (vs. just the first and second finger present or not a finger in sight).

The candidate new species differs from 15 congeners by lacking vomerine teeth, with a vomerine ridge present. It differs from *B. daiyunensis*, *B. daweimontis*, *B. elongata*, *B. fansipanensis*, *B. frigida*, *B. hoanglienensis*, *B. jinggangensis*, *B. jiulianensis*, *B. nanlingensis*, *B. palpebralespinosa*, *B. pepe*, *B. rubrimera*, *B. shimentaina*, *B. tongboensis*, and *B. yingdeensis* (vs. presence of vomerine teeth). The candidate new species differs from the following nine congeners by lacking lateral fringes on its toes: *B. anlongensis*, *B. baishanzuensis*, *B. binchuanensis*, *B. congjiangensis*, *B. lini*, *B. xiangnanensis*, *B. xianjuensis*, *B. yangmingensis* (vs. presence of lateral fringes on the toes in these species), and *B. wushanensis* (vs. presence of wide lateral fringes on the toes in males, lacking in females).

Compared to other congeners, The candidate new species has a smaller body size, with an SVL of 37.6–40.2 mm (38.9 ± 1.3, *n* = 7) in adult males and an SVL of 41.8–45.9 mm (43.7 ± 2.1, *n* = 3) in adult females. It is significantly different from the nine congeners whose adult male and female SVL is ≥ 50 mm, including *B*. *caudoprocta* (81.3 mm in males), *B.fangjingmontis* (58.6–63.8 in males), *B*. *jingdongensis* (53.0–56.5 mm in males), *B*. *liboensis* (60.5–67.7 mm in males), *B*. *mirabilis* (55.8–61.4 mm in males), *B*. *omeimontis* (56.0–59.5 mm in males), *B*. *sangzhiensis* (53.0–60.8 mm in male), *B*. *shuichengensis* (102.0–118.3 mm in males), and *B*. *spinata* (47.2–54.4 mm in males). The candidate new species can be further distinguished from other congeners by possessing two subarticular tubercles at the base of each finger (vs. three metacarpal tubercles found in *B.jiangi*), and having tibio-tarsal articulation reaching forward between the anterior margin of the tympanum and the posterior corner of the eye (vs. tibio-tarsal articulation reaching to the tip of the snout in *B*. *shunhuangensis*, or the middle of the eye in *B. caobangensis*).

### 3.3. Taxonomic Account

Based on the results of these molecular phylogenetic and morphological comparisons, the specimen is distinct from all other congeners of *Boulenophrys*. The description of this new species is provided below.

#### *Boulenophrys dupanglingensis* Xiao & Mo, sp. nov.

Dupangling Horned Toad (in English)/(都庞岭角蟾 in Chinese)

https://zoobank.org/pub:AF74BC71-7B79-4FC7-8B18-F373D3609648

**Figure 4:**
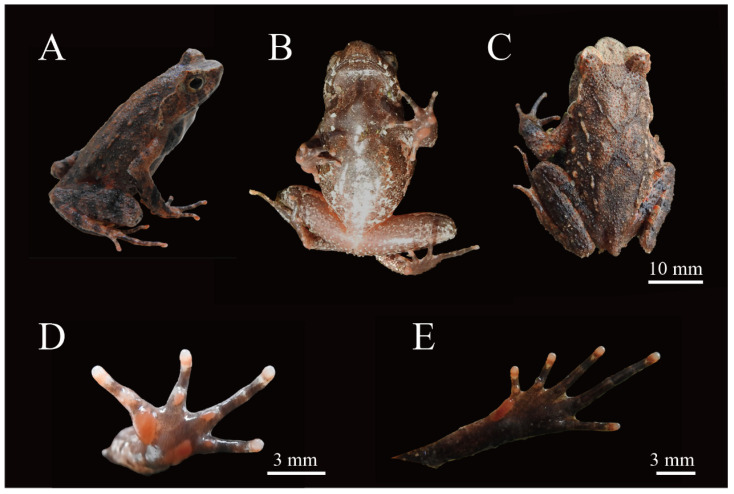
Holotype of *Boulenophrys dupanglingensis* sp. nov. (HUNU 22SA01) in life: (**A**) lateral view; (**B**) ventral view; (**C**) dorsal view; (**D**) volar view of left hand; (**E**) plantar view of left foot. Photo by Jia-Yan Xi.

**Figure 5:**
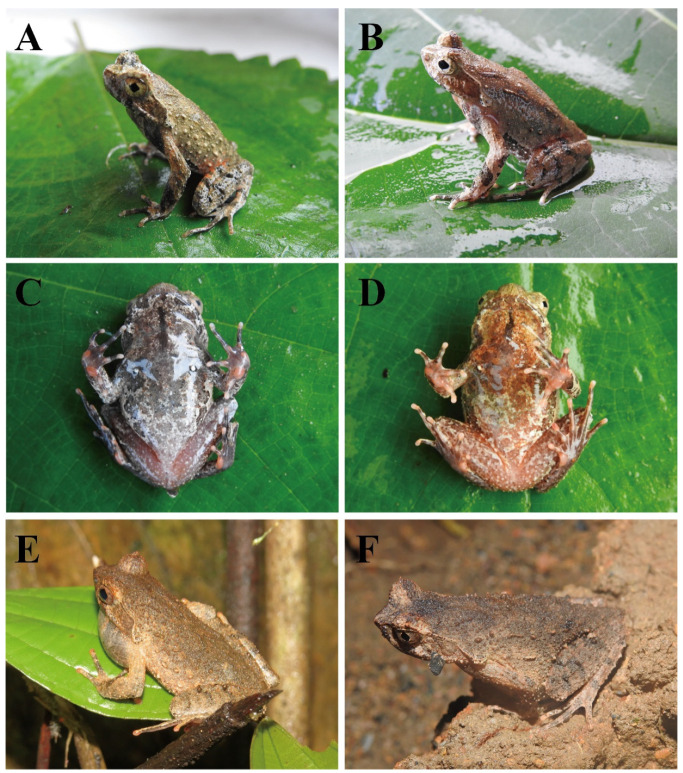
Paratypes of *Boulenophrys dupanglingensis* sp. nov. in life. Male paratype (HUNU 22SA003): (**A**,**C**,**E**); female paratype (HUNU 22SA009): (**B**,**D**,**F**). Photos by Jia-Yan Xi.

Figure 4 and Figure 5

****

Holotype. Adult male, HUNU 22SA01 (Figure 4), collected by Xiaoyang Mo on 20 April 2022 from Dajiangyuan Village (111.35931958° E, 25.46065718° N; 380 m a.s.l.), Dupangling National Nature Reserve, Daoxian County, Yongzhou City, Hunan Province, China.

Paratypes: Nine adults: five adult males (HUNU 22SA02–06) were collected from the same locality as the holotype on 21 April 2022 and 23 June 2016 by Xiaoyang Mo, Bin Wang, Jiayan Xi, Qi Li, Hui Li, Leqiang Zhu, Ayinuer Maimaiti, Yalan Xu, and Fan Zhou., one adult male CIB 2016050802 was collected from type locality on 8 May 2016 by Shengchao Shi; one adult female (HUNU 22SA07), which was collected from Daguyuan Village, Jiangyong County (111.33171300° E, 25.43785510° N; 442 m a.s.l.) on 23 April 2022 (Figure 5) by Xiaoyang Mo, Bin Wang, Jiayan Xi,; two adult females (HUNU 22SA08–09), which were collected from Daguyuan Village, Jiangyong County (112.24011945° E, 25.33178657° N; 451 m a.s.l.) on 24 April 2022 by Hui Li, Leqiang Zhu, Ayinuer Maimaiti.

Etymology: The specific epithet *dupanglingensis* refers to the type locality of the new species, Dupangling National Nature Reserve, Hunan Province, China. Dupangling Horned Toad is suggested as common name. 都庞岭角蟾 (dū pánɡ lǐnɡ jiǎo chán) is suggested as Chinese name.

**Figure 6:**
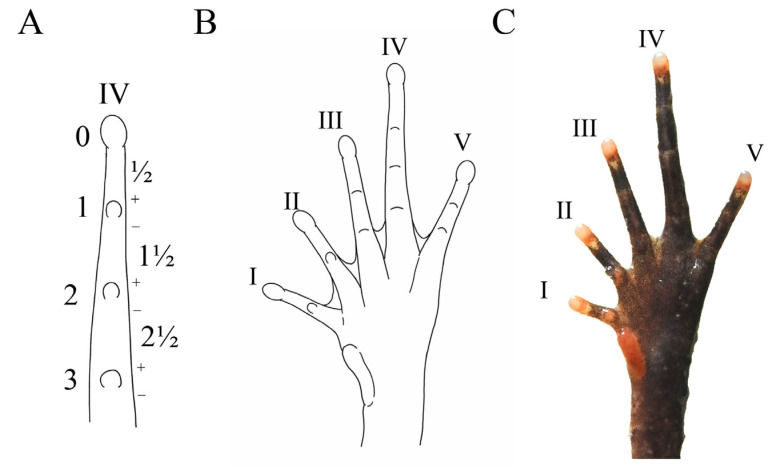
Description of webbing formula according to Savage and Heyer (1997). (**A**) Pattern of the fourth toes finger, with phalangeal joint used as the measurement; (**B**) pattern of the ventral foot in *B. dupanglingensis* sp. nov.; (**C**) photograph of the ventral foot. Photo by Jia-Yan Xi.

**Figure 7:**
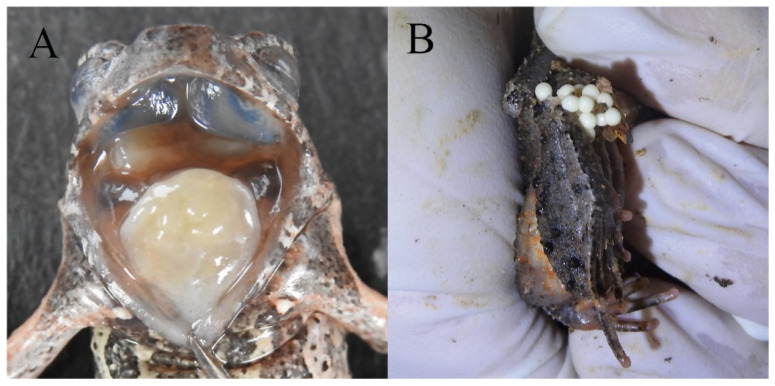
(**A**) Edge of the tongue; (**B**) eggs of living paratype. Photo by Jia-Yan Xi.

Diagnosis: (1) moderate body size: SVL 37.6–40.2 mm (38.9 ± 1.3, *n* = 7) in adult males and SVL 41.8–45.9 mm (43.6 ± 2.1, *n* = 3) in adult females; (2) tympanum boundary clear: TD/ED 0.48–0.57 in males, 0.47–0.57 in females; (3) presence of small horn-like tubercle at the edge of the upper eyelid; (4) vomerine ridge present; vomerine teeth absent; (5) margin of tongue rounded, not notched posteriorly; (6) rough dorsal skin: a discontinuous “V”-shaped ridge with two discontinuous dorsolateral ridges on two sides on the back, dense tubercles on the skin of the ventral surface of the dorsal shank and thigh, and spiny tubercles surrounding the cloaca; (7) slender hindlimbs with heels overlapping when the flexed hindlimbs are held at right angles to the body axis; tibio-tarsal articulation reaching forward between anterior margin of tympanum and posterior corner of the eye when leg stretched forward; (8) relative finger length IV < II < I < III, with a subarticular tubercle present at the base of each finger; (9) distinct supernumerary tubercles below the base of toes I and II; (10) toes without lateral fringes and with only rudimentary webbing (webbing formula: I1 − 1-II1 − 2-III2 − 3IV3- − 2V) (Figure 6 and Figure 7A).

Coloration of holotype in life. Forearms and hindlimbs are marked with dark-brown transverse bands; supratympanic ridges are light ivory with a dark spot on the posterior edge; a dark vertical band extends from the lower margin of the eye to the upper lip; numerous brown patches are scattered across the lateroventral surface of the flanks; the groin is red-orange; the ventral surface of the throat and chest is light salmon, with dark-brown patches and dark-orange blotches; distinct longitudinal stripe along the throat; ventral body surface is light salmon in color with brown patches and white spots; ventral surfaces of limbs are light salmon with dark-brown spots and blotches; hands and feet have brown ventral surfaces, with pale brown tips on the digits; metacarpal and metatarsal tubercles are reddish; pectoral glands and femoral glands appear white; iris is yellowish brown.

Coloration of holotype in preservative. Dark-brown coloration has faded to greyish brown dorsally. A triangular marking is visible between the eyes, accompanied by a “V”-shaped marking along the mid-dorsum; transverse bands on dorsal sides of the forearms and hindlimbs have become indistinct. The ventral surface has faded to a greyish white color, with all previously distinct bands and spots becoming less noticeable or completely indistinguishable.

Variation and sexual dimorphism. Measurement data for the type series are provided in Table 2. Morphological diagnostic characters observed in all paratypes were consistent with those of the holotype. However, there were variations in coloration and stripe patterns between individuals (Figure 4). For example, the adult male paratype (HUNU 22SA03) with a smaller body size (SVL 37.8 mm) has a light-brown back and a black-brown belly, the ventral surfaces of the limbs are primarily black-brown, and there is a prominent black spot at the right upper eyelid. In contrast, the adult female paratype (HUNU 22SA09) with a larger body size (SVL 41.8 mm) has a yellowish-brown dorsal coloration, the chest and anterior abdomen exhibit dark-orange blotches on a light-brown background, the ventral surfaces of the limbs are predominantly orange-red, and fewer markedly enlarged tubercles are present on the flanks.

Sexual size dimorphism: Females (SVL 43.6 ± 2.1 mm, *n* = 3) are significantly larger than males (SVL 38.8 ± 0.9 mm, *n* = 6). Adult males possess a single internal subgular vocal sac. During the breeding season, males develop prominent black nuptial spines at the bases of the first and second fingers on dorsal surfaces.

Reproductive characteristics: The eggs are creamy white, with an approximate diameter of 2.6 mm. The observed clutch size consisted of approximately 258 eggs (female, HUNU 22SA08, Figure 7B)

**Figure 8:**
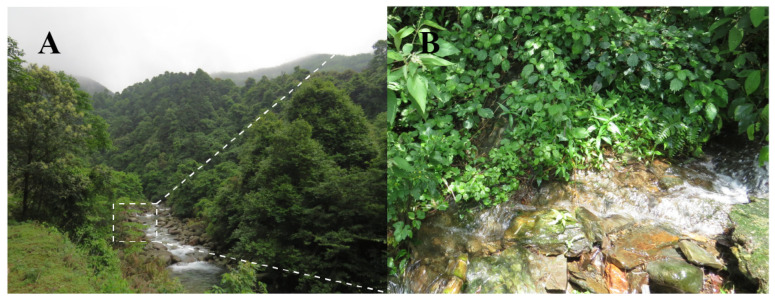
Habitat of *Boulenophrys dupanglingensis* sp. nov.(**A**) Landscape of montane forests at the type locality. (**B**) microhabitats of the new species, mountain stream (white box shown in the Fig 8A). Photo by Sheng-Chao Shi.

Distribution and habitats. *Boulenophrys dupanglingensis* sp. nov. is only found in the Dupangling National Nature Reserve, located in southern Hunan, China. The species was observed in evergreen secondary forest, typically near mountain streams, where it was found among the leaf litter on the forest floor. The elevation records of the new species range from 380 to 451 m (Figure 8). Calls of males were recorded between April and June, suggesting that the species’ breeding season including this period of time. Additionally, female specimens collected during this time were found to contain mature, creamy white eggs. No tadpoles were encountered during the field surveys.

## 4. Discussion

The discover and description the new species once more highlight the diversity of *Boulenophrys*, which is currently classified into three primary subgroups: the *B. boettgeri* group, the *B. minor* group, and the *B. omeimontis* group [10,23,24]. The newly described species, *Boulenophrys dupanglingensis*, is classified within the *B. boettgeri* group. This new species brings the total number of recognized *Boulenophrys* species in Hunan Province to 10, including *B. caudoprocta*, *B. dupanglingensis*, *B.hengshanensi*, *B. mufumontana*, *B.Sangzhiensis*, *B. shunhuangensis*, *B. tuberogranulatus*, *B. xiangnanensis*, *B. xuefengmontis*, and *B. yangmingensis* [10,25,26], and it suggests that further investigation on this group might lead to more findings of hidden diversity.

Most species of *Boulenophrys* are endemic to small region [5,10], although *B. dupanglingensis* and *B. yunkaiensis* exhibit a close evolutionary relationship, yet their habitats are separated by low lands in southern Hunan province (Figure 1). The Nanling Mountain range spans the provinces of Guangxi, Hunan, Guangdong, and Jiangxi, forming a natural barrier separating the Yangtze River and Pearl River basins. This region exhibits species diversity, strong forest dependence, and overlapping distributions of the *Boulenophry* genus [27]. These patterns highlight the need for further research into speciation processes, niche differentiation, and coexistence mechanisms [28]. Moreover, climatic fluctuations, habitat diversity, sexual selection, and the dynamics of montane forests likely drive diversification within *Boulenophrys*. Geographic isolation often leads to allopatric speciation, while ecological factors can drive adaptive divergence, influencing the evolution of complex phenotypes. For further studies, it would be valuable to analysis skeletal and acoustic features, which could provide insights into the evolution and speciation of this group.

The new species was found to be abundant at its habitat in breeding seasons from 2015 to 2022 based on our field investigation. However, considering the endemism of the genus, the distribution area of this species is expected to restricted to small region bordering Hunan and Guangxi. And this species is expected to found in Guilin of northeastern Guangxi. The known distribution area is located in the Dupangling National Nature Reserve and the habitat is well protected. The conservation status for this species is suggested to be evaluated based on further investigation covering all its distribution area. 

## 5. Conclusions

Combining the molecular phylogenetic and morphological analysis results, a new *Boulenophry* species, *B*. *dupanglingensis* sp. nov., is described from Yongzhou City, Hunan Province, China. This research brings the total number of recognized *Boulenophrys* species in Hunan Province to 10. This discovery highlights the biodiversity within the Nanling Mountain range and further emphasizes the importance of the region as a biodiversity hotspot.

## Figures and Tables

**Table 1:** Diagnostic characters distinguishing all 69 species of *Boulenophrys*. For species with unavailable data, “/” is indicated.

	Species	SVL in Males (in mm)	SVL in Females (in mm)	Horn-like Tubercle at Upper Eyelid: Larger (T); Smaller (F)	Vomerine Teeth: Present (T); Absent (F)	Tongue: Notched (T); Not Notched (F)	Heels Overlapping: (T) Just Meeting (N); Not Meeting (F)	Lateral Fringes on Toes: Wide (T); Narrow (N); Absent (F)	Webs on Toes: More Than One-Fourth (T); Rudimentary (N); Lacking (F)
1	*B*. *dupanglingensis* sp. nov.	37.8–40.2	41.8–45.9	F	F	F	T	F	N
2	*B*. *acuta*	27.1–33.0	28.1–33.6	T	F	F	F	N	N
3	*B*. *angka*	31.2–32.1	37.5–39.2	F	F	F	T/N	F	N
4	*B*. *anlongensis*	40.0–45.5	48.9–51.2	F	F	F	T	N	N
5	*B*. *baishanzuensis*	28.4–32.4	/	F	F	F	T	N	F
6	*B*. *baolongensis*	42.0–45.0	/	F	F	T	N	F	F
7	*B*. *binchuanensis*	32.0–36.0	40.2–42.5	F	F	T/F	N	T	N
8	*B*. *binlingensis*	45.1–51.0	/	F	F	T	T	/	N
9	*B*. *boettgeri*	34.5–37.8	39.7–46.8	F	F	T	N	T	N
10	*B*. *brachykolos*	33.7–39.3	33.9–45.9	F	F	F	F	F	N
11	*B*. *caobangensis*	34.9–38.9	/	F	F	F	/	F	N
12	*B*. *caudoprocta*	81.3	/	T	T	F	N	/	N
13	*B*. *congjiangensis*	28.6–33.4	38.4–40.2	F	F	F	T	N	N
14	*B*. *cheni*	26.2–29.5	31.8–34.1	F	F	T	T	T	N
15	*B*. *chishuiensis*	43.4–44.1	44.8–49.8	F	F	F	T	F	N
16	*B*. *daiyunensis*	27.6–28.7	33.7–35.6	F	T	F	T/N	N	N
17	*B*. *daoji*	32.6–33.6	37.5–41.4	F	F	F	F	N	N
18	*B*. *daweimontis*	34.0–37.0	40.0–46.0	F	T	/	/	F	F
19	*B*. *dongguanensis*	30.2–39.3	/	F	T	F	F	F	N
20	*B. elongata*	28.2–28.5	35.1–37.6	F	T	F	N	N	F
21	*B. fanjingmontis*	58.2–63.6	62.8–72.2	F	T	T	N	T	N
22	*B*. *fansipanensis*	30.9–44.3	41.7–42.5	F	T	T/F	/	F	F
23	*B*. *fengshunensis*	34.3–39.4	42.5–44.9	F	T	F	F	F	N
24	*B*. *frigida*	30.3–31.8	/	F	T	T/F	/	F	F
25	*B.hengshanensis*	35.7–41.2	37.5–50.2	F	F	F	F	F	F
26	*B. hoanglienensis*	37.4–47.6	59.6	F	T	T	/	F	N/F
27	*B*. *hungtai*.	25.8–33.3	/	F	F	F	F	F	F
28	*B*. *insularis*	36.8–41.2	47.1	F	T	T	F	F	N
29	*B*. *jiangi*	34.4–39.2	39.5–40.4	F	F	F	T	F	N
30	*B*. *jingdongensis*	53.0–56.5	63.5	F	T	T	T	T	T
31	*B*. *jinggangensis*	35.1–36.7	38.4–41.6	T	T	F	T	N	N
32	*B*. *jiulianensis*	30.4–33.9	34.1–37.5	F	T	T	T	F	N
33	*B*. *kuatunensis*	26.2–31.4	26.6–37.3	F	F	T	T/F	F/N	F
34	*B*. *leishanensis*	30.4–38.7	42.3	F	F	F	T	F	N
35	*B. lichun*	33.5–37.0	47.1	F	T	F	F	F	F
36	*B*. *liboensis*	60.5–67.7	60.8–70.6	T	T	T	T	T	N
37	*B*. *lini*	34.1–39.7	37.0–39.9	F	F	F	T	T	N
38	*B*. *lishuiensis*	30.7–34.7	36.9–40.4	F	F	F	/	F	F
39	*B*. *lushuiensis*	31.0–34.8	/	F	F	T	/	N	N
40	*B*. *minor*	34.5–41.2	/	F	F	T	T/N	F	N
41	*B*. *mirabilis*	55.8–61.4	68.5–74.8	T	F	F	T	N	N
42	*B*. *mufumontana*	30.1–30.8	36.3	F	F	F	T	N	N
43	*B*. *nankunensis*	29.9–34.9	39.4–41.9	F	T	F	F	F	N
44	*B*. *nanlingensis*	30.5–37.3	/	F	T	T	T	N	N
45	*B*. *obesa*	35.6	37.5–41.2	F	F	F	F	F	N
46	*B*. *ombrophila*	27.4–34.5	32.8–35.0	F	F	F	F	F	F
47	*B*. *omeimontis*	56.0–59.5	68.0–72.5	F	T	T	T	N	N
48	*B*. *palpebralespinosa*	36.2–38.0	/	T	T	F	T	T	T
49	*B. pepe*	35.3–36.4	/	F	F	T	F	F	F
50	*B*. *puningensis*	31.7–34.6	37.8–38.3	F	T	F	F	F	N
51	*B*. *qianbeinsis*	49.3–58.2	/	F	T	T	T	T	T
52	*B*. *rubrimera*	26.6–30.8	/	F	T	T/F	/	N	F
53	*B*. *sangzhiensis*	54.7	/	F	T	T	T	N	N
54	*B*. *sanmingensis*	27.0–29.5	29.5	F	F	T	T	T	N
55	*B*. *shimentaina*	28.0–30.6	/	F	T	F	T	N	N
56	*B*. *shuichengensis*	102.0–118.3	99.8–115.6	T	F	T	/	T	T
57	*B*. *shunhuangensis*	30.3–33.7	37.6	F	F	F	T	F	F
58	*B*. *spinata*	47.2–54.4	54.0–55.0	F	F	T	T	T	T
59	*B*. *tongboensis*	26.5–31.5	/	F	T	T	T	F	F
60	*B*. *tuberogranulatus*	33.2–39.0	50.5	F	F	F	/	F	N
61	*B*. *wugongensis*	31.0–34.1	38.5–42.8	F	F	F	F	F	N
62	*B*. *wuliangshanensis*	27.3–31.6	41.3	F	F	T/F	T	F	F
63	*B*. *wushanensis*	30.4–35.5	38.4	F	F	F	F	F/T	T
64	*B*. *xiangnanensis*	38.6–42.0	44.4	F	F	F	N	T	N
65	*B*. *xianjuensis*	31.0–36.3	41.6	F	F	F	T	N	N
66	*B. xuefengmontis*	37.0–38.3	45.3–48.9	F	F	F	T/N	F	F
67	*B*. *yaoshanensis*	32.5–42.6	46.6–47.4	F	F	F	T/N	F	N
68	*B*. *yangmingensis*	33.2–37.1	45.2	F	F	F	T	N	N
69	*B*. *yingdeensis*	33.2–35.3	36.3–45.8	F	T	F	T/N	F	N
70	*B*. *yunkaiensis*	35.3–40.0	45.3–46.1	F	F	F	T/N	F	N

**Table 2:** Measurements (in mm) and ratios for the type series of *Boulenophrys dupanglingensis* sp. nov. “*” denotes the holotype.

	HUNU 22SA01 *	HUNU 22SA02	HUNU 22SA03	HUNU 22SA04	HUNU 22SA05	HUNU 22SA06	CIB2016050802	HUNU 22SA07	HUNU 22SA08	HUNU 22SA09
Sex	Male	Male	Male	Male	Male	Male	Male	Female	Female	Female
SVL	39.6	38.1	37.8	40.2	38.8	38.5	37.6	43.0	45.9	41.8
HDL	13.3	12.8	13.2	12.1	12.1	12.1	11.3	13.3	13.1	13.2
HDW	13.7	13.2	13.4	12.7	13.4	13.2	12.6	14.2	14.9	14.0
SNT	3.3	3.3	3.2	3.4	3.2	3.1	4.4	3.2	3.4	3.1
IND	4.4	4.1	3.7	4.2	4.2	3.9	4.2	4.4	4.7	4.4
IOD	3.1	3.3	3.3	3.7	3.2	3.3	3.8	3.6	3.8	3.7
ED	6.3	5.6	5.2	6.0	5.6	5.8	4.2	5.5	6.0	5.3
TD	3.3	3.2	2.9	2.9	3.1	3.1	2.3	2.6	3.1	3.0
TED	1.8	1.8	1.8	1.8	1.4	1.6	2.4	2.0	2.5	2.1
HND	9.7	9.2	9.7	9.5	9.2	9.2	8.4	10.3	11.8	9.8
RAD	8.0	7.4	7.8	7.7	7.6	7.2	8.9	8.0	8.3	7.1
TIB	15.1	14.8	14.9	15.5	14.8	14.4	14.6	15.7	18.6	15.8
FTL	23.8	23.1	23.2	23.6	23.2	22.8	/	25.4	27.3	26.7
TD/ED	0.52	0.57	0.48	0.48	0.55	0.53	0.54	0.53	0.47	0.57
RAD/SVL	0.20	0.19	0.21	0.19	0.20	0.19	0.24	0.19	0.18	0.17
TIB/SVL	0.38	0.39	0.39	0.39	0.38	0.37	0.39	0.37	0.41	0.38
FTL/SVL	0.60	0.61	0.61	0.59	0.60	0.59	/	0.59	0.59	0.64

## Data Availability

The original contributions presented in the study are included in the article/Supplementary Materials, further inquiries can be directed to the corresponding author.

## Supplementary Materials

The following supporting information can be downloaded at: https://www.mdpi.com/article/10.3390/ani15030440/s1, Table S1: Information on the morphological characters of 69 recognized species of *Boulenophrys*. Table S2: Localities, voucher information, and GenBank accession numbers for all samples used in this study. Table S3: Uncorrected *p*-distance (%) among *Boulenophrys* species inferred from mitochondrial *COI* gene [29,30,31,32,33,34,35,36,37,38,39,40,41,42,43,44,45,46,47,48,49,50,51,52,53,54,55,56,57,58,59,60,61].
