# Supplementary material for: A New Species of the Genus Boulenophrys (Anura, Megophryidae) from Southern Hunan Province, Central China
Source: Animals (Basel). 2025 Feb 5;15(3):440. doi: 10.3390/ani15030440 (PMC11816057; doi:10.3390/ani15030440)
Supplement: Supplementary file 1 [file animals-15-00440-s001.zip › animals-3395367-supplementary/supplementary materials/supplementary Table S1.pdf]

supplementary materials

Table S1. Literature for morphological characters of 69 recognized species of *Boulenophrys*.

ID	***Boulenophrys*** **species**	**References**
1	*B. acuta* (Wang, Li & Jin, 2014)	Li et al. 2014[1]
2	*B. angka* (Wu, Suwannapoom, Poyarkov, Pawangkhanant, Xu, Jin, Murphy & Che, 2019)	Wu et al. 2019[2]
3	*B. anlongensis* (Li, Lu, Liu & Wang, 2020)	Li et al. 2020a[3]
4	*B. baishanzuensis* (Wu, Li, Liu, Wang & Wu, 2020)	Wu et al. 2020[2]
5	*B. baolongensis* (Ye, Fei & Xie, 2007)	Ye et al. 2007[4]
6	*B. binchuanensis* (Ye & Fei, 1995)	Fei and Ye 2016[5]
7	*B. binlingensis* (Jiang, Fei & Ye, 2009)	Fei and Ye 2016[5]
8	*B. boettgeri* (Boulenger, 1899)	Fei and Ye 2016[5]
9	*B. brachykolos* (Inger & Romer, 1961)	Fei and Ye 2016[5]
10	*B. caobangensis* (Nguyen, Pham, Nguyen, Luong & Ziegler, 2020)	Nguyen et al. 2020[6]
11	*B. caudoprocta* (Shen, 1994)	Fei and Ye 2016[7]
12	*B. congjiangensis* (Luo, Wang, Wang, Lu, Wang, Deng & Zhou, 2021)	Luo et al. 2021[8]
13	*B. cheni* (Wang & Liu, 2014)	Wang et al. 2014[9]
14	*B. chishuiensis* (Xu, Li, Liu, Wei & Wang, 2020)	Xu et al. 2020[10]
15	*B. daiyunensis* (Lyu, Wang & Wang, 2021)	Lyu et al. 2021[11]
16	*B. daoji* (Lyu, Zeng, Wang & Wang, 2021)	Lyu et al. 2023[12]
17	*B. daweimontis* (Rao & Yang, 1997)	Fei & Ye 2016[5]
18	*B. dongguanensis* (Wang & Wang, 2019)	Wang et al. 2019a[13]
19	*B. elongata* (Zeng, Wang, Chen, Xiao, Zhan, Li & Lin 2024)	Zeng et al. 2024[14]
20	*B. fanjingmontis* (Zhang, Liang, Ran & Shen 2012)	Zhang et al.2012[15]
21	*B. fansipanensis* (Tapley, Cutajar, Mahony, Nguyen, Dau, Luong, Le, Nguyen, Nguyen, Portway, Luong & Rowley, 2018)	Tapley et al. 2018[16]
22	*B. fengshunensis* (Wang, Zeng, Lyu, & Wang, 2022)	Wang et al. 2022[17]
23	*B. frigida* (Tapley, Cutajar, Nguyen, Portway, Mahony, Nguyen, Harding, Luong & Rowley, 2021)	Tapley et al. 2020[18]
24	*B. hengshanensis* (Qian, Hu, Mo, Gao, Zhang, & Yang, 2023)	Qian et al. 2023[19]
25	*B. hoanglienensis* (Tapley, Cutajar, Mahony, Nguyen, Dau, Luong, Le, Nguyen, Nguyen, Portway, Luong & Rowley, 2018)	Tapley et al. 2018[20]
26	*B. hungtai* (Wang, Zeng, Lyu, Xiao, & Wang, 2022)	Wang et al. 2022[17]
27	*B. insularis* (Wang, Liu, Lyu, Zeng & Wang, 2017)	Wang et al. 2017a[21]
28	*B. jiangi* (Liu, Li, Wei, Xu, Cheng, Wang & Wu, 2020)	Liu et al. 2020[22]
29	*B. jingdongensis* (Fei & Ye, 1983)	Fei & Ye 2016[5]
30	*B. jinggangensis* (Wang, 2012)	Wang et al. 2012[23]
31	*B. jiulianensis* (Wang, Zeng, Lyu & Wang, 2019)	Lyu et al. 2023[12]
32	*B. kuatunensis* (Pope, 1929)	Fei & Ye 2016; [5]

ID	*Boulenophrys* species	References
33	*B. leishanensis* (Li, Xu, Liu, Jiang, Wei & Wang, 2018)	Li *et al*. 2018[24]
34	*B.lichun* (Lin, Chen, Li, Peng, Zeng &Wang, 2024)	Lin *et al*. 2024[25]
35	*B. lushuiensis* (Shi, Li, Zhu, Jiang, Jiang & Wang, 2021)	Shi *et al*. 2021[26]
36	*B. liboensis* (Zhang, Li, Xiao, Li, Pan, Wang, Zhang & Zhou, 2017)	Zhang *et al*. 2017[27]
37	*B. lini* (Wang & Yang, 2014)	Wang *et al*. 2014[9]
38	*B. lishuiensis* (Wang, Liu & Jiang, 2017)	Wang *et al*. 2017b[28]
39	*B. minor* (Stejneger, 1926)	Fei & Ye 2016[5]
40	*B. mirabilis* (Lyu, Wang & Zhao, 2020)	Lyu *et al*. 2020[29]
41	*B. mufumontana* (Wang, Lyu & Wang, 2019)	Wang *et al*. 2019a[13]
42	*B. nankunensis* (Wang, Zeng & Wang, 2019)	Wang *et al*. 2019a[13]
43	*B. nanlingensis* (Lyu, Wang, Liu & Wang, 2019)	Wang *et al*. 2019a[13]
44	*B. obesa* (Wang, Li & Zhao, 2014)	Li *et al*. 2014[1]
45	*B. ombrophila* (Messenger & Dahn, 2019)	Messenger *et al*. 2019[30]
46	*B. omeimontis* (Liu, 1950)	Fei & Ye 2016[5]
47	*B. palpebralespinosa* (Bourret, 1937)	Fei & Ye 2016[5]
48	*B. pepe* (Wang & Zeng, 2024)	Wang et al. 2024[31]
49	*B. puningensis*（Wang, Zeng, Lyu, Xiao, & Wang, 2022）	Wang *et al*. 2022[17]
50	*B. qianbeinsis* (Su, Shi, Wu, Li, Yao, Wang & Li, 2020)	Su *et al*. 2020[32]
51	*B. rubrimera* (Tapley, Cutajar, Mahony, Chung, Dau, Nguyen, Luong & Rowley, 2017)	Tapley *et al*. 2017[33]
52	*B. sangzhiensis* (Jiang, Ye & Fei, 2008)	Jiang *et al*. 2008[34]
53	*B. sanmingensis* (Lyu & Wang, 2021)	Lyu *et al*. 2023[11]
54	*B. shimentaina* (Lyu, Liu & Wang, 2020)	Lyu *et al*. 2023[12]
55	*B. shuichengensis* (Tian and Sun, 1995)	Tian *et al*. 2000[35]
56	*B. shunhuangensis* (Wang, Deng, Liu, Wu & Liu, 2019)	Wang *et al*. 2019b[28]
57	*B. spinata* (Liu & Hu, 1973)	Fei & Ye 2016[5]
58	*B. tongboensis* (Wang & Lyu, 2021)	Lyu *et al*. 2023[12]
59	*B. tuberogranulatus* (Shen, Mo & Li, 2010)	Mo *et al*. 2010[36]
60	*B. wugongensis* (Wang, Lyu & Wang, 2019)	Wang *et al*. 2019a[13]
61	*B. wuliangshanensis* (Ye & Fei, 1995)	Fei and Ye 2020[37]
62	*B. wushanensis* (Ye & Fei, 1995)	Fei and Ye 2020[37]
63	*B. xiangnanensis* (Lyu, Zeng & Wang, 2020)	Lyu *et al*. 2023[12]
64	*B. xianjuensis* (Wang, Wu, Peng, Shi, Lu & Wu, 2020)	Wang *et al*. 2020[38]
65	*B. xuefengmontis* (Lyu & Wang, 2023)	Lyu et al. 2023[12]
66	*B. yangmingensis* (Lyu, Zeng & Wang, 2020)	Lyu *et al*. 2023[12]
67	*B. yaoshanensis* (Qi, Mo, Lyu, Wang & Wang, 2021)	Qi *et al*. 2021[39]
68	*B. yingdeensis* (Qi, Lyu, Wang & Wang, 2021)	Qi *et al*. 2021[39]
69	*B. yunkaiensis* (Qi, Wang, Lyu & Wang, 2021)	Qi *et al*. 2021[39]

Reference

1. Li, Y.L., Jin, M. J., Zhao, J., Liu, Z. Y., Wang, Y. Y., Pang, H. Description of two new species of the genus Megophrys (Amphibia: Anura: Megophryidae) from Heishiding Nature Reserve, Fengkai, Guangdong, China, based on molecular and morphological data. *Zootaxa* **2014**, *3795*, 449-471, doi:10.11646/zootaxa.3795.4.5.
2. Wu, Y.H., Suwannapoom, C., Poyarkov, N. A., Jr., Pawangkhanant, P., Xu, K., Jin, J. Q., Murphy, R. W., Che, J. A new species of the genus Xenophrys Anura Megophryidae from northern Thailand. *Zoological Research* **2019**, *40*, 564-574, doi:10.24272/j.issn.2095-8137.2019.032.
3. Li, Y., Zhang, D.D., Lyu, Z.T., Wang, J., Li, Y. L., Liu, Z. Y., Chen, H.H., Rao, D.Q., Jin, Z.F., Zhang, C.Y., Wang, Y.Y. Review of the genus Brachytarsophrys (Anura: Megophryidae), with revalidation of Brachytarsophrys platyparietus and description of a new species from China. *Zoological Research* **2020**, *41*, 105-122, doi:10.24272/j.issn.2095-8137.2020.033.
4. Ye, C.Y., Fei, L., Xie, F. A new species of Megophryidae–Megophyrys baolongensis from China (Amphibia, Anura). *Acta Herpetologica Sinica* **2007**, *11*, 38-41.
5. Fei, L., Ye, C. Y. *Amphibians of China*; Chengdu Institute of Biology, Chinese Academy of Sciences. Science Press: Beijing, China, 2016; Volume 1.
6. Nguyen, T.Q., Pham, C. T., Nguyen, T. T., Luong, A. M., Ziegler, T. A new species of Megophrys (Amphibia: Anura: Megophryidae) from Vietnam. *Zootaxa* **2020**, *4722.5.1*, 401-422, doi:10.11646/zootaxa.4722.5.1.
7. Shen, Y.H., Yang, D. D., Mo, X. Y., Li, H. H., Chen, D. *The Fauna of Hunan: Amphibia*; Hunan Science and Technology Press: Changsha, 2014.
8. Luo, T., Wang, Y., Wang, S., Lu, X., Wang, W., Deng, H., Zhou, J. A species of the genus Panophrys (Anura, Megophryidae) from southeastern Guizhou Province, China. *Zookeys* **2021**, *1047*, 27-60, doi:10.3897/zookeys.1047.61097.
9. Wang, Y.Y., Zhao, J., Yang, J.H., Zhou, Z., Chen, G.L., Liu, Y. Morphology, molecular genetics, and bioacoustics support two new sympatric Xenophrys toads (Amphibia: Anura: Megophryidae) in southeast China. *PLoS One* **2014**, *9*, e93075, doi:10.1371/journal.pone.0093075.
10. Xu, N., Li, S. Z., Liu, J., Wei, G., Wang, B. A new species of the horned toad Megophrys Kuhl & Van Hasselt, 1822 (Anura, Megophryidae) from southwest China. *Zookeys* **2020**, *943*, 119-144, doi:10.3897/zookeys.943.50343.
11. Lyu, Z.T., Zeng, Z. C., Wang, J., Liu, Z. Y., Huang, Y. Q., Li, W. Z., Wang, Y. Y. Four new species of Panophrys (Anura, Megophryidae) from eastern China, with discussion on the recognition of Panophrys as a distinct genus. *Zootaxa* **2021**, *4927*, 009-040, doi:10.11646/zootaxa.4927.1.2.
12. Lyu, Z.T., Qi, S., Wang, J. Zhang, S. Y., Zhao, J., Zeng, Z. C., Wan, H., Yang, J. H., Mo, Y. M., Wang, Y. Y. Generic classification of Asian horned toads (Anura: Megophryidae: Megophryinae) and monograph of Chinese species. *Zoological Research* **2023**, *44*, 380-450, doi:10.24272/j.issn.2095-8137.2022.372.
13. Wang, J., Lyu, Z. T., Liu, Z. Y., Liao, C. K., Zeng, Z. C., Zhao, J., Li, Y. L., Wang, Y. Y. Description of six new species of the subgenus Panophrys within the genus Megophrys (Anura, Megophryidae) from southeastern China based on molecular and morphological data. *Zookeys* **2019a**, *851*, 113-164, doi:10.3897/zookeys.851.29107.
14. Zeng, Z.C., Wang, J., Chen, H.H., Xiao, W.W., Zhan, B.B., Li, Y.H., Lin, S.S. A New Species of the Genus <italic>Boulenophrys</italic> (Anura, Megophryidae) from Eastern

Guangdong, China. *Asian Herpetological Research* **2024**, *15*, 12-21, doi:10.3724/ahr.2095-0357.2023.0038.

15. Zhang, L., Liang, L.,Ran, H., Shen, Z.X. Megophrys binlingensis fanjingmontis: A New Subspecies of Megophryidae from Guizhou, China. *Chinese Journal of Zoology* **2012**, *47*, 135-138, doi:10.13859/j.cjz.2012.04.020.
16. Tapley, B., Cutajar, T., Nguyen, L.T., Nguyen, C.T., Harding, L., Portway, C., Van Luong, H. & Rowley, J.J. A new locality and elevation extension for Megophrys rubrimera (Tapley et al., 2017) in Bat Xat Nature Reserve, Lao Cai Province, northern Vietnam. *Herpetology Notes* **2018b**, *11*, 865-868.
17. Wang, J., Zeng, Z. C., Lyu, Z. T., Qi, S., Liu, Z. Y., Chen, H. H., Lu, Y. H.,; Xiao, H.W., Lin, C. R., Chen, K., Wang, Y. Y. Description of three new Boulenophrys species from eastern Guangdong, China, emphasizing the urgency of ecological conservation in this region (Anura, Megophryidae). *Zootaxa* **2022**, *5099*, 91-119, doi:10.11646/zootaxa.5099.1.4.
18. Tapley, B., Cutajar, T., Nguyen, L. T., Portway, C.,Mahony, S., Nguyen, C.T.,Harding, L.,Luong, H.V., Rowley, J. L. A new potentially Endangered species of Megophrys (Amphibia: Megophryidae) from Mount Ky Quan San, north-west Vietnam. *Journal of Natural History* **2020**, *54*, 2543-2575, doi:10.1080/00222933.2020.1856952.
19. Qian, T.Y., Hu, K., Mo, X.Y., Gao, Z.W., Zhang, N., Yang, D.D. A new species of Boulenophrys from central Hunan Province, China (Anura: Megophryidae). *Vertebrate Zoology* **2023**, *73*, 915-930, doi:10.3897/vz.73.e100889.
20. Tapley, B., Cutajar, T., Mahony, S., Nguyen, C.T., Dau, V.Q., Luong, A.M., Le, D.T., Nguyen, T.T., Nguyen, T.Q., Portway, C., Luong, H. V., Rowley, J. L. Two new and potentially highly threatened Megophrys Horned frogs (Amphibia: Megophryidae) from Indochina's highest mountains. *Zootaxa* **2018a**, *4508*, 301-333, doi:10.11646/zootaxa.4508.3.1.
21. Wang, J.; Liu, Z.Y.; Lyu, Z.T.; Zeng, Z.C.; Wang, Y.Y. A new species of the genus Xenophrys (Amphibia: Anura: Megophryidae) from an offshore island in Guangdong Province, southeastern China. *Zootaxa* **2017a**, *4324*, 541-556, doi:10.11646/zootaxa.4324.3.8.
22. Liu, J., Li, S. Z., Wei, G., Xu, N., Cheng, Y. L., Wang, B., Wu, J. A New Species of the Asian Toad Genus Megophrys sensu lato (Anura: Megophryidae) from Guizhou Province, China. *Asian Herpetological Research* **2020**, *11*, 1-18,18A-18I, doi:10.16373/j.cnki.ahr.190041.
23. Wang, Y.Y., Zhang, T. D., Zhao, J., Sung, Y. H., Yang, J. H., Pang, H., ; Zhang, Z. Description of a new species of the genus Xenophrys Gunther, 1864 (Amphibia: Anura: Megophryidae) from Mount Jinggang, China, based on molecular and morphological data. *Zootaxa* **2012**, *3546*, 53-67.
24. Li, S.Z.; Xu, N.; Liu, J.; Jiang, J.P.; Wei, G.; Wang, B. A New Species of the Asian Toad Genus Megophrys sensu lato (Amphibia: Anura: Megophryidae) from Guizhou Province, China. *Asian Herpetological Research* **2018**, *9*, 224-239E, doi:10.16373/j.cnki.ahr.180072.
25. Lin, S.S., Chen, H. H., Li, Y. H., Peng, Z. N., Zeng, Z. C., Wang, J. A new Boulenophrys species (Anura, Megophryidae) from the coastal hills of eastern Fujian Province, China. *Zookeys* **2024**, *1216*, 1-15, doi:10.3897/zookeys.1216.130017.
26. Shi, S.C., Li, D.H., Zhu, W.B., Wang, J., Jiang, J.P., Ye, C.Y., Fei, L., Wang, B. Description of a new toad of Megophrys Kuhl & Van Hasselt, 1822 (Amphibia: Anura: Megophryidae) from western Yunnan Province, China. *Zootaxa* **2021**, *4942*, 351-381, doi:10.11646/zootaxa.4942.3.3.
27. Zhang, Y.N., Li, G., Xiao, N., Li, J.Q., Pan, T., Wang, H., Zhang, B.W., Zhou, J. A New Species of

the Genus Xenophrys (Amphibia: Anura: Megophryidae) from Libo County, Guizhou, China. *Asian Herpetological Research* **2017**, *8*, 75-85, doi:10.16373/j.cnki.ahr.160041.
28. Wang, L., Deng, X.J., Liu, Y., Wu, Q.Q., Liu, Z. A new species of the genus Megophrys (Amphibia: Anura: Megophryidae) from Hunan, China. *Zootaxa* **2019b**, *4695*, 301-330, doi:10.11646/zootaxa.4695.4.1.
29. Lyu, Z.T., Li, Y.Q., Zeng, Z.C., Zhao, J., Liu, Z.Y., Guo, G.X., Wang, Y.Y. Four new species of Asian horned toads (Anura, Megophryidae, Megophrys) from southern China. *ZooKeys* **2020**, *942*, 105-140, doi:10.3897/zookeys.942.47983.
30. Messenger, K.R., Dahn, H.A., Liang, Y., Xie, P., Wang, Y., Lu, C. A new species of the genus Megophrys Gunther, 1864 (Amphibia: Anura: Megophryidae) from Mount Wuyi, China. *Zootaxa* **2019**, *4554*, 561-583, doi:10.11646/zootaxa.4554.2.9.
31. Wang, J., Lin, S.S., Gan, J.S., Chen, H.H., Yu, L.M., Pan, Z., Xiao, J.J., Zeng, Z.C. A new species of the genus Boulenophrys from South China (Anura, Megophryidae). *Zootaxa* **2024**, *5514*, 451-468, doi:10.11646/zootaxa.5514.5.3.
32. Su, H.J., Shi, S.C., Wu, Y.Q., Li, G.R., Yao, X.G., Wang, B., Li, S.Z. Description of a new horned toad of Megophrys Kuhl & Van Hasselt, 1822 (Anura, Megophryidae) from southwest China. *Zookeys* **2020**, 131-159, doi:10.3897/zookeys.974.56070.
33. Tapley, B., Cutajar, T., Mahony, S., Nguyen, C. T., Dau, V. Q., Nguyen, T. T., Luong, H. V., Rowley, J. J. L. The Vietnamese population of Megophrys kuatunensis (Amphibia: Megophryidae) represents a new species of Asian horned frog from Vietnam and southern China. *Zootaxa* **2017**, *4344*, 465-492, doi:10.11646/zootaxa.4344.3.3.
34. Jiang, J.P., Ye, C.Y., Fei, L. A New Horn Toad Megophrys sangzhiensis from Hunan, China (Amphibia, Anura). *Zoological Research* **2008**, *29*, 219-222.
35. Tian, Y.Z., Gu, X. M., Sun, A. Q. A new species of Xenophrys in China (Amphibia: Pelobatidae). *Acta Zootaxonomica Sinica* **2000**, *25*, 462-466.
36. Mo, X.Y., Shen, Y.H., Li, H.H., Wu, X.S. A new species of Megophrys (Amphibia: Anura: Megophryidae) from the northwestern Hunan Province, China. *Current Zoology* **2010**, *56*, 432-436, doi:10.1093/czoolo/56.4.432.
37. Fei, L. *Atlas of Amphibians in China. Field Edition*; Henan Science and Technology Press: Zhengzhou, p. 432.
38. Wang, B., Wu, Y. Q., Peng, J. W., Shi, S. C., Lu, N. N., Wu, J. A new Megophrys Kuhl & Van Hasselt (Amphibia, Megophryidae) from southeastern China. *Zookeys* **2020**, *904*, 35-62, doi:10.3897/zookeys.904.47354.
39. Qi, S., Lyu, Z. T., Wang, J. Mo, Y. M., Zeng, Z. C., Zeng, Y. J., Dai, K. Y., Li, Y. Q., Grismer, L. L., Wang, Y. Y. Three new species of the genus Boulenophrys (Anura, Megophryidae) from southern China. *Zootaxa* **2021**, *5072*, 401-438, doi:10.11646/zootaxa.5072.5.1.
