# Supplementary material for: A New Species of the Genus Boulenophrys (Anura, Megophryidae) from Southern Hunan Province, Central China
Source: Animals (Basel). 2025 Feb 5;15(3):440. doi: 10.3390/ani15030440 (PMC11816057; doi:10.3390/ani15030440)
Supplement: Supplementary file 1 [file animals-15-00440-s001.zip › animals-3395367-supplementary/supplementary materials/Supplementary TableS2.pdf]

Table S2. Sample list of genus *Boulenophrys* and outgroups for phylogenetic analysis in this study.

ID	Species	Voucher ID	Localities	16S	COI
In-groups: *Boulenophrys*					
1	*B. dupanglingensis* sp. nov.	HUNU 22SA05	Dupangling National Nature Reserve, Hunan, China	OP548599	OP550328
2	*B. dupanglingensis* sp. nov.	HUNU 22SA07	Dupangling National Nature Reserve, Hunan, China	OP548597	OP550329
3	*B. dupanglingensis* sp. nov.	HUNU 22SA08	Dupangling National Nature Reserve, Hunan, China	OP548598	OP550330
4	*B. dupanglingensis* sp. nov.	CIB2016050802	Dupangling National Nature Reserve, Hunan, China	PQ569619	/
5	*B. acuta*	SYS a002276	Heishiding, Guangdong, China	KJ579124	MH406126
6	*B. angka*	KIZ 040592	Kiew Mae Pan Nature Trail, Doi Inthanon, Chiang Mai, Thailand	MN508048	/
7	*B. anlongensis*	CIB AL20190531018	Anlong County, Guizhou, China	MT823184	MT823261
8	*B. baishanzuensis*	QY 20200726001	Qingyuan County, Zhejiang, China	MW001155	MT998296
9	*B. baolongensis*	KIZ 019216	Baolong, Chongqing, China	KX811813	KX812093
10	*B. binchuanensis*	KIZ 019441	Jizu Shan, Yunnan, China	KX811849	KX812112
11	*B. binlingensis*	SYS a005313	Wawu Shan, Sichuan, China	MH406892	MH406354
12	*B. boettgeri*	SYS a004149	Mt. Wuyi, Fujian, China	MF667878	MH406247
13	*B. brachykolos*	SYS a002258	Hong Kong, China	KJ560403	MH406120
14	*B. caobangensis*	IBER 4385	Nguyen Binh, Cao Bang, Vietnam	LC483945	/
15	*B. caudoprocta*	SYS a004281	Zhangjiajie, Hunan, China	MH406795	MH406257
16	*B. cheni*	SYS a002126	Taoyuandong, Hunan, China	MH406659	MH406096
17	*B. chishuiensis*	CIB CS20190518031	Chishui City, Guizhou, China	MN954707	MN928958
18	*B. congjiangensis*	GZNU 20200706003	Yueliangshan Nature Reserve, Congjiang, Guizhou, China	MW959773	MW959761
19	*B. daiyunensis*	SYS a001733	Daiyun Mountain Nature Reserve, Fujian, China	MH406643	MH406079
20	*B. daoji*	SYS a006212	Mt Tiantai, Zhejiang, China	MW367047	MW365497
21	*B. daweimontis*	KIZ 048997	Mt Dawei, Yunnan, China	KX811867	KX812125
22	*B. dongguanensis*	SYS a001973	Mt Yinping, Guangdong, China	MH406647	MH406083
23	*B.elongata*	GEP a150	Huidong County, Huizhou City, Guangdong, China	OR601592	OR597098
24	*B. fansipanensis*	AMS R186115	Sa Pa, Lao Cai, Vietnam	MH514887	MW086548
25	*B. fanjingmontis*	SYS a004350	China, Guizhou, Mt Fanjing, China	MH406808	MH406270

ID	Species	Voucher ID	Localities	16S	COI
26	*B. fengshunensis*	SYS a004724	Fengshun County, Guangdong, China	MH406848	MH406310
27	*B. frigida*	AMS R186131	Mount Ky Quan San, Bat Xat District, Lao Cai, Vietnam	MT364279	MW086550
28	*B.hengshanensis*	CSUFT HS210608	Mt. Hengshan, Nanyue Dist., Hengyang, Hunan, China	ON209289	OQ910104
29	*B. hoanglienensis*	VNMN 2018.02	Sa Pa, Lao Cai, Vietnam	MH514889	MW086551
30	*B. hungtai*	SYS a007575	Jiexi County, Guangdong, China	OL635592	OL634859
31	*B. insularis*	SYS a002169	Nan'ao Island, Guangdong, China	MH406663	MH406103
32	*B. jiangi*	CIB 180722006	Kuankuoshui Nature Reserve, Guizhou, China	MN107743	MN107748
33	*B. jingdongensis*	SYS a003928	Mt. Wuliang, Yunnan, China	MH406773	MH406232
34	*B. jinggangensis*	SYS a004824	Mt. Sifang, Hunan, China	MH406857	MH406319
35	*B. jiulianensis*	SYS a004219	Mt Jiulian, Jiangxi, China	MH406791	MH406253
36	*B. kuatunensis*	SYS a003449	Guadun, Fujian, China	MF667881	MH406206
37	*B.lichun*	GEP a211	Mt Nanji, Fujian, China	PQ309138	PQ300666
38	*B. leishanensis*	SYS a002213	Mt. Leigong, Guizhou, China	MH406673	MH406113
39	*B. liboensis*	GNUG 20150813001	Libo Country, Guizhou, China	MF285253	/
40	*B. lini*	SYS a002381	Mt. Jinggang, Jiangxi, China	MF667874	MH406135
41	*B. lishuiensis*	WYF 00169	Lishui, Zhejiang, China	KY021418	/
42	*B. lushuiensis*	SYS a003823	Lushui County, Yunnan, China	MH406763	MH406222
43	*B. minor*	SYS a003209	Dujiangyan, Sichuan, China	MF667862	MH406194
44	*B. mirabilis*	SYS a002917	Huaping Nature Reserve, Guangxi, China	MH406724	MH406176
45	*B. mufumontana*	SYS a006390	Mt Mufu, Hunan, China	MK524104	MK524135
46	*B. nankunensis*	SYS a004501	Mt Nankun, Guangdong, China	MH406822	MH406284
47	*B. nanlingensis*	SYS a001964	Nanling Nature Reserve, Guangdong, China	MH406646	MH406082
48	*B. obesa*	SYS a002271	Heishiding, Guangdong, China	KJ579121	MH406123
49	*B. ombrophila*	WUYI 2015101	Mt Wuyi, Fujian, China	KX856397	/
50	*B. omeimontis*	KIZ 025765	Emei Shan, Sichuan, China	KX811884	KX812136
51	*B. palpebralespinosa*	KIZ 011650	Pu Hu Nature Reserve, Thanh Hoa, Vietnam	KX811889	KX812138
52	*B.pepe*	GEP a207	Qingyuan, Guangdong, China	PQ131151	PQ130479
53	*B. puningensis*	SYS a005770	Puning City, Guangdong, China	OL635585	OL634853

ID	Species	Voucher ID	Localities	16S	COI
54	*B. qianbeiensis*	CIBTZ 20190608017	Tongzi County, Guizhou, China	MT651554	MT654521
55	*B. rubrimera*	VNMN 2017.002	Sa Pa, Lao Cai, Vietnam	MF536420	/
56	*B. sangzhiensis*	SYS a004306	Zhangjiajie, Hunan, China	MH406797	MH406259
57	*B. sanmingensis*	SYS a002499	Mt Longqi, Fujian, China	MH406699	MH406147
58	*B. shimentaina*	SYS a004173	Shimentai Nature Reserve, Guangdong, China	MH406788	MH406250
59	*B. shuichengensis*	TRUtrxy01	Shuicheng, Guizhou,China	/	/
60	*B. shunhuangensis*	HUNU 18NS01	Nanshan Forest Park, Hunan, China	MK836023	MK977594
61	*B. spinata*	SYS a002226	Mt. Leigong, Guizhou, China	MH406675	MH406115
62	*B. tongboensis*	SYS a003225	Mt Tongbo, Jiangxi, China	MH406742	MH406199
63	*B. tuberogranulatus*	SYS a004310	Zhangjiajie, Hunan, China	MH406801	MH406263
64	*B. wugongensis*	SYS a004800	Mt Wugong, Jiangxi, China	MH406853	MH406315
65	*B. wuliangshanensis*	SYS a003924	Mt. Wuliang, Yunnan, China	MH406771	MH406230
66	*B. wushanensis*	SYS a003008	Mt. Wu, Hubei, China	MH406732	MH406184
67	*B. xiangnanensis*	SYS a002875	Mt. Yangming, Hunan, China	MH406714	MH406166
68	*B. xianjuensis*	CIB XJ190503	Xianju County, Zhejiang, China	MN563758	MN563774
69	*B. xuefengmontis*	SYS a004364	Wugang County, Hunan, China	MH406813	MH406275
70	*B. yangmingensis*	SYS a002890	Mt. Yangming, Hunan, China	MH406721	MH406173
71	*B. yaoshanensis*	SYS a002189	Dayaoshan Nature Reserve, Guangxi, China	MH406667	MH406107
72	*B. yaoshanensis*	SYS a002190	Dayaoshan Nature Reserve, Guangxi, China	MH406668	MH406108
73	*B. yaoshanensis*	SYS a003548	Dayaoshan Nature Reserve, Guangxi, China	OK491615	OK493609
74	*B. yingdeensis*	SYS a002100	Shimentai Nature Reserve, Guangdong, China	MH406658	MH406095
75	*B. yunkaiensis*	SYS a004637	Yunkaishan Nature Reserve, Guangdong, China	MH406843	MH406305
76	*B. yunkaiensis*	SYS a004638	Yunkaishan Nature Reserve, Guangdong, China	MH406844	MH406306
77	*B. yunkaiensis*	SYS a004694	Yunkaishan Nature Reserve, Guangdong, China	MH406845	MH406307
Out-groups: *Xenophrys*					
78	*X. glandulosa*	SYS a003758	Mt. Gaoligong, Yunnan, China	MH406755	MH406214
79	*X. mangshanensis*	SYS a002177	Mt. Sanyue, Guangdong, China	MH406666	MH406106
